# Potential applications of a new short food frequency questionnaire for CKD patients

**DOI:** 10.1186/s12882-017-0765-5

**Published:** 2017-11-27

**Authors:** Deidra C. Crews

**Affiliations:** 1 Division of Nephrology, Department of Medicine, Johns Hopkins University School of Medicine, 301 Mason F. Lord Drive, Suite 2500, Baltimore, MD 21224 USA; 2 Welch Center for Prevention, Epidemiology and Clinical Research, Johns Hopkins Medical Institutions, Baltimore, MD USA; 3 Center on Aging and Health, Johns Hopkins Medical Institutions, Baltimore, MD USA; 4 Johns Hopkins Center for Health Equity, Johns Hopkins Medical Institutions, Baltimore, MD USA

## Abstract

Diet is among the most modifiable risk factors for chronic kidney disease (CKD) progression. Studies of beneficial dietary patterns for persons with CKD have largely utilized dietary assessment measures developed in individuals without CKD. In *BMC Nephrology*, Affret et al. report on their study of the reproducibility of a newly developed short 49-item food frequency questionnaire (SFFQ) adapted to patients with CKD. Here, we discuss potential applications of this SFFQ, towards better understanding of which dietary patterns portend the most favorable outcomes in CKD.

Diet is one of the most modifiable risk factors for adverse health outcomes, including progressive chronic kidney disease (CKD). Numerous randomized controlled trials have evaluated single nutrient targets (i.e. protein or sodium intake) for their impact on CKD outcomes [1]. Dietary patterns have also been examined, largely in the setting of observational studies, for their relation to CKD outcomes. Several recent studies have applied measures ascertaining accordance to healthful dietary patterns such as the Dietary Approaches to Stop Hypertension (DASH) and Mediterranean diets to understand their relation to kidney outcomes [2]. Investigators have also built upon guidelines for healthful eating in the general population to examine how these dietary patterns impact risk of CKD and rapid kidney function decline [3]. These measures and guidelines, however, have not been developed in persons with CKD—limiting their application in this population.

In *BMC Nephrology*, Affret et al. report on their study of the reproducibility of a newly developed short 49-item food frequency questionnaire (SFFQ) adapted to patients with CKD [4]. They evaluated the feasibility and validity of the SFFQ in the CKD-REIN (CKD-Renal Epidemiology and Information Network) which is a prospective cohort of adults with moderate or advanced CKD (stages 3 to 4) drawn from a nationally representative sample of nephrology facilities in France [5]. The authors used a food list for the SFFQ that was developed based upon national food questionnaires and individual food intakes of French adults. The first portion of the SFFQ included 40 food group items, while the second portion included 9 questions focused on persons with CKD. These CKD-related questions estimated intake of protein, calcium, phosphorus, potassium and sodium, and included questions about added salt during cooking and processed food consumption. To validate the findings of the SFFQ, the investigators administered six 24-h dietary recalls over the course of the year between the first and the follow up SFFQ and compared these data to the SFFQs. They found acceptable relative validity and reproducibility including a demonstration of the ability to rank participants appropriately for nutrients of interest for persons with CKD.

If modified and validated in other settings where commonly consumed foods may differ from those in France, the SFFQ developed by Affret et al. has several potential applications relevant to existing knowledge gaps in nutrition and CKD. First, questions still remain regarding which dietary patterns lead to favorable outcomes in CKD. While some researchers have focused on overall dietary patterns using indices developed in general (largely free of CKD) populations, few have applied these to examine long-term outcomes among persons with CKD [6–8]. For example, a dietary pattern characterized by fried foods, organ meats, and sweetened beverages, which is commonly consumed in the Southern U.S., was associated with higher mortality in a study of persons with CKD [7]. In the same study, a plant-based dietary pattern high in fruits, vegetables and fish was associated with lower mortality; and none of the dietary patterns examined predicted ESRD risk [7]. A limitation of most observational studies of this kind is that dietary patterns are only measured once (i.e. at study baseline) or twice [9] and are then analyzed for their relation to CKD outcomes occurring years later. While understudied in CKD, dietary patterns may change over time, especially as CKD progresses. For those persons aware of their CKD and under the care of a nephrologist, for example, they may be advised to reduce consumption of certain nutrients (e.g. phosphorus). In advanced CKD, reduced appetite may lead to a change in dietary pattern. Understanding the longitudinal relationships of dietary patterns with CKD outcomes is an important area worthy of investigation, and an opportunity to apply repeated measures of a SFFQ.

Another potential application of the SFFQ by Affret et al. would be to examine whether certain dietary patterns predict favorable outcomes in specific kidney diseases. One could envisage, for example, that individuals with proteinuric disease might respond differently to a dietary pattern characterized by reduced sodium than would those with tubulo-interstitial disease. There have been several dietary intervention studies targeting specific CKD populations, such as diabetic kidney disease and hypertension-attributed nephropathy [1], yet the overall dietary patterns predictive of favorable outcomes in the setting of specific kidney diseases are poorly understood. Elucidating these patterns could advance our understanding of potential nutritional intervention targets in CKD. For example, in the report by Affret et al., a total of 37.8% and 39.3% of participants in the validity and reproducibility studies, respectively, had diabetes [biopsy-proven causes of kidney disease in the study were not noted] [4]. Their hemoglobin A1C values were controlled, at 6.3% in both groups. Whether persons with diabetes and CKD had dietary patterns that varied from those without diabetes was not detailed in the study, but could be a valuable future application of the SFFQ especially if related to clinical outcomes.

Diet is but one health behavior important for kidney health. Others, such as physical activity and avoidance of tobacco, may also be important contributors. A SFFQ specific to persons with CKD could be useful in the setting of cohort studies to determine the relative contribution of dietary patterns to CKD progression among other health behaviors. Rebholz et al. [10] recently reported on the American Heart Association ‘Life’s Simple 7’ metric for cardiovascular health promotion in a study of CKD risk. The authors found that risk of incident CKD was inversely related to the number of ideal health factors. Individually, they found that smoking, body mass index, physical activity, blood pressure, and blood glucose were associated with CKD risk, however diet and blood cholesterol were not. This lack of association between a healthy diet and CKD risk may have been due to the specific dietary factors and thresholds (e.g. sodium < 1500 mg/day) used to define a heart healthy diet not being comparably relevant for kidney disease risk [10]. Notably, Affret et al. found significant differences between sodium intakes as estimated by 24-h recalls (5058.4 mg/day) as compared to the second SFFQ (2319.3 mg/day) [4]. The relative contribution of sodium intake as compared to other health behaviors among persons with CKD, would be an interesting investigation, for which a SFFQ might be applied.

Socially disadvantaged groups are at increased risk for CKD progression, and dietary factors may be contributors. For example, food insecurity (“limited or uncertain ability to acquire nutritionally adequate and safe foods in socially acceptable ways”) is associated with risk of CKD progression among U.S. adults [11]. Upstream from CKD progression, it has been shown that persons living in poverty have dietary patterns that are less ‘healthful’ than those not living in poverty, and this correlates with impoverished persons’ greater prevalence of CKD [12]. Further understanding of the dietary patterns of food insecure and other disadvantaged persons with CKD could inform tailored interventions for these high-risk populations.

Clinicians may use questionnaires as tools in their dietary counseling of patients with CKD. While the 49-item SFFQ by Affret et al. is quite comprehensive, and potentially useful in research settings, shorter screeners could be used clinically. For example, consumption of fruits and vegetables, which correlates with lower dietary acid load (a risk factor for CKD progression [6]) can be assessed using very short tools. A two-question measure for fruit and vegetable intake developed and used in a British study by Wardle et al. [13] and validated by Capuccio et al. [14] asks: “1. How many pieces of fruit, of any sort, do you eat on a typical day?” and “2. How many portions of vegetables, excluding potatoes, do you eat on a typical day?” These questions identify more than 80% of individuals with biomarker profiles indicative of low fruit and vegetable intake (i.e., consuming less than five portions of fruits and vegetables per day) [14], which may contribute to CKD progression.

There have been few dietary intervention studies adequately powered to examine CKD progression [1]. The repeated use of a SFFQ developed in persons with CKD offers opportunities to assess whether change in dietary pattern is possible, and whether it correlates with clinical markers of kidney health. Such interventions would be best tailored to patient populations at highest risk for CKD progression, including socially disadvantaged groups.
